# Association of Educational, Occupational and Socioeconomic Status with Cardiovascular Risk Factors in Asian Indians: A Cross-Sectional Study

**DOI:** 10.1371/journal.pone.0044098

**Published:** 2012-08-29

**Authors:** Rajeev Gupta, Prakash C. Deedwania, Krishnakumar Sharma, Arvind Gupta, Soneil Guptha, Vijay Achari, Arthur J. Asirvatham, Anil Bhansali, Balkishan Gupta, Sunil Gupta, Mallikarjuna V. Jali, Tulika G. Mahanta, Anuj Maheshwari, Banshi Saboo, Jitendra Singh, Rajiv Gupta

**Affiliations:** 1 Department of Medicine, Fortis Escorts Hospital, Jaipur, Rajasthan, India; 2 Jaipur Heart Watch Foundation, Jaipur, Rajasthan, India; 3 Department of Cardiology, University of California San Francisco and Veterans’ Administration Hospital, Fresno, California, United States of America; 4 Jaipur Diabetes Research Centre, Jaipur, Rajasthan, India; 5 Department of Medicine, Patna Medical College, Patna, Bihar, India; 6 Department of Medicine, Arthur Asirvatham Hospital, Madurai, Tamilnadu, India; 7 Department of Endocrinology, Postgraduate Institute of Medical Education and Research, Chandigarh, India; 8 Department of Medicine, Sardar Patel Medical College, Bikaner, Rajasthan, India; 9 Diabetes Care and Research Centre, Nagpur, Maharashtra, India; 10 Department of Medicine, Jawaharlal Nehru Medical College, Belgaum, Karnataka, India; 11 Department of Community Medicine, Assam Medical College, Dibrugarh, Assam, India; 12 Department of Medicine, Babu Banarasi Das College of Dental Sciences, Lucknow, Uttar Pradesh, India; 13 DiaCare and Research, Ahmadabad, Gujarat, India; 14 Department of Medicine, Government Medical College, Jammu, Jammu and Kashmir, India; 15 Department of Sociology, University of Rajasthan, Jaipur, Rajasthan, India; University of Pittsburgh Medical Center, United States of America

## Abstract

**Background:**

To determine correlation of multiple parameters of socioeconomic status with cardiovascular risk factors in India.

**Methods:**

The study was performed at eleven cities using cluster sampling. Subjects (n = 6198, men 3426, women 2772) were evaluated for socioeconomic, demographic, biophysical and biochemical factors. They were classified into low, medium and high socioeconomic groups based on educational level (<10, 10–15 and >15 yr formal education), occupational class and socioeconomic scale. Risk factor differences were evaluated using multivariate logistic regression.

**Results:**

Age-adjusted prevalence (%) of risk factors in men and women was overweight or obesity in 41.1 and 45.2, obesity 8.3 and 15.8, high waist circumference 35.7 and 57.5, high waist-hip ratio 69.0 and 83.8, hypertension 32.5 and 30.4, hypercholesterolemia 24.8 and 25.3, low HDL cholesterol 34.1 and 35.1, high triglycerides 41.2 and 31.5, diabetes 16.7 and 14.4 and metabolic syndrome in 32.2 and 40.4 percent. Lifestyle factors were smoking 12.0 and 0.5, other tobacco use 12.7 and 6.3, high fat intake 51.2 and 48.2, low fruits/vegetables intake 25.3 and 28.9, and physical inactivity in 38.8 and 46.1%. Prevalence of > = 3 risk factors was significantly greater in low (28.0%) vs. middle (23.9%) or high (22.1%) educational groups (p<0.01). In low vs. high educational groups there was greater prevalence of high waist-hip ratio (odds ratio 2.18, confidence interval 1.65–2.71), low HDL cholesterol (1.51, 1.27–1.80), hypertriglyceridemia (1.16, 0.99–1.37), smoking/tobacco use (3.27, 2.66–4.01), and low physical activity (1.15, 0.97–1.37); and lower prevalence of high fat diet (0.47, 0.38–0.57),overweight/obesity (0.68, 0.58–0.80) and hypercholesterolemia (0.79, 0.66–0.94). Similar associations were observed with occupational and socioeconomic status.

**Conclusions:**

Low educational, occupational and socioeconomic status Asian Indians have greater prevalence of truncal obesity, low HDL cholesterol, hypertriglyceridemia, smoking or tobacco use and low physical activity and clustering of > = 3 major cardiovascular risk factors.

## Introduction

India is changing rapidly [1], [2]. The World Bank has reclassified the country from low-income to lower-middle income group indicating increase in national income, increased per capita income and per capita expenditure, and better social and human development indices [3]. Concurrently the disease patterns have also changed [4], [5]. From predominantly communicable, nutritional, maternal and childhood causes of morbidity and mortality in the last century, there has been a shift to preponderance of non-communicable diseases as major causes of disability and deaths [5]. This could be due to social and economic changes that lead to disease transition from communicable to non-communicable diseases [5], [6]. Similar changes occurred in countries of Europe and North America about a hundred years ago [6]–[9]. In socioeconomically primitive societies, chronic disease risk factors such as smoking, physical inactivity, obesity, hypertension, hypercholesterolemia and diabetes are more in high socioeconomic status subjects [8]. With social and economic development and ongoing epidemiological transition in these countries the risk factors become more prevalent among the lower socioeconomic status subjects [8]. For example, in UK in early and mid twentieth century, cardiovascular risk factors and cardiovascular diseases were more common in upper and middle social classes as compared to the lower [9]. Reduction of risk factors among the higher social classes, due to greater awareness and better treatments, led to decline in cardiovascular disease incidence and mortality in higher socioeconomic groups [9], [10]. In the lower socioeconomic groups the risk factors did not change, decline of cardiovascular mortality was absent or slow [9]. In these countries the cardiovascular diseases prevalence is currently more among low socioeconomic classes than in the higher socioeconomic class [10].

Influence of socioeconomic status on non-communicable disease risk factors, especially cardiovascular risk factors, has not been well studied in India [11]. Epidemiological studies in mid- and late-twentieth century reported greater cardiovascular risk factor prevalence among the higher social classes [12], [13]. At the turn of the century there appeared a transition in risk factors and studies that correlated educational status, as a marker of socioeconomic status, reported greater prevalence of risk factors such as smoking and hypertension among less educated rural subjects and industrial populations [14]–[16]. Studies have also reported greater Framingham Risk Score among the illiterate and low educational status subjects [16]–[18]. However, there are no studies that have evaluated influence of multiple markers of socioeconomic status (education, occupation or social status) on prevalence of cardiovascular risk factors. Therefore, to determine prevalence of cardiovascular risk factors among urban Indian populations and to study influence of multiple markers of socioeconomic status on cardiovascular risk we performed a nationwide cross sectional epidemiological study. The study evaluated risk factors among the urban middle class which is the biggest subset of Indian population [2]. This group of subjects provides unique opportunity to identify influence of microlevel social factors (education, occupation, etc.) on cardiovascular risk. We did not include rural or very low socioeconomic status urban subjects because in these populations there could be many other sociobiological confounders that influence cardiovascular risk [9], [10]. Study among this subset of Indian population is also important because majority of the Indian population shall reside in urban locations by middle of the century [2].

## Methods

A nationwide study to identify prevalence of cardiovascular risk factors and their sociodemographic determinants was organised among urban subjects in India. Rationale for the study has been reported earlier [19]. Protocol was approved by the institutional ethics committee of the national coordinating centre (Fortis Escorts Hospital, Jaipur, India; US-Federal Wide Assurance Number 00017762). Written informed consent was obtained from each participant. No minor (<18 years age) participated in the study. The study case report form was developed according to recommendations of the World Health Organization [20] and was modified from the form used previously in our studies [21], [22].

### Regions and Investigators

We planned the study according to the regional variations in cardiovascular mortality reported by Registrar General of India [19], [23], [24]. Medium sized cities were identified in each of the large states of India and investigators who had a track record of research in cardiovascular or diabetes epidemiology were invited for participation in the study. 20 investigators were invited from all large states of India and 15 agreed to participate. The cities were in northern (Jammu, Chandigarh, Karnal, Bikaner), western (Ahmadabad, Jaipur), eastern (Lucknow, Patna, Dibrugarh), southern (Madurai, Hyderabad, Belgaum) and central (Indore, Nagpur) regions of India. A steering committee and investigators meeting was organised at initiation of the study where the study protocol was discussed and developed. The meeting was followed by training in salient features of questionnaire and techniques of examination and evaluation to ensure uniformity in recruitment and data collection. Later, 4 investigators dropped out due to non-availability of technical support and motivation and 11 finally performed the survey. The combined population of these eleven cities according to the 2001 Indian census was 21 million [25]. There is a middle class of about 30% in these cities [2] and, therefore, the sampled locations represent a population of about 7 million subjects and 5 million adults.

### Sampling

Simple cluster sampling was performed at each site. A middle-class location was identified at each city. This is based on municipal classification and is derived from cost of land, type of housing, public facilities (roads, sanitation, water supply, electricity, gas supply, etc.), educational and medical facilities [26]. A sample size of about 250 men and 250 women (n = 500) at each site is considered adequate by World Health Organization (WHO) to identify 20% difference in mean level of biophysical and biochemical risk factors [20]. We invited 800–1000 subjects in each location to ensure participation of at least 500 subjects at each site estimating a response of 70% as reported in previous studies at similar locations [21]. Target sample sizes at some sites was decreased due to low recruitments (e.g., Belgaum), and increased at high recruiting sites (e.g., Jaipur, Madurai). Oversampling was performed at some sites to have uniform regional representation. At each site a uniform protocol of recruitment was followed. Accordingly, a locality within the urban area of the city was identified, houses enumerated, number of subjects >18–75 years living in each house determined and all these individuals were invited to a local community centre or healthcare facility (clinic, dispensary) for examination and blood investigations. This procedure ensured participation of consecutive members of the locality and was representative even if the survey was prematurely abandoned at a particular location (e.g., Belgaum, Nagpur). This method also ensured representativeness at sites where oversampling was performed. The surveys were preceded by meetings with community leaders to ensure good participation. Subjects were invited in fasting state to a community/medical centre within each locality either twice or thrice a week depending upon the investigator’s schedule. Inclusion criteria were all adults aged >18–75 years living in the particular location. Subjects who were confined to home with severe debilitating disease, those not likely to survive beyond 6 months and pregnant women were excluded.

### Measurements

The study case report form was filled by the research worker after details were inquired from the subject. Apart from demographic history, details of socioeconomic status based on educational status and years of formal education, occupational class (British Social Register classification, six categories) [9] as well as self assessed socioeconomic status (10 category scale; 1 being lowest and 10 highest) [27], were inquired. Smoking details were inquired for type of smoking or non-smoked tobacco use, number of cigarettes/bidis smoked and years of smoking. Intake of alcohol was assessed as drink per week, dietary fat was assessed using questions about type of cooking oil used and estimated as daily visible fat intake (g). Fruits and vegetables intake were assessed by a question that inquired number of servings (medium portions) of either fruits or green leafy vegetables per day. Details of physical activity were assessed by questions for exact daily duration (minutes) of work related-, commute related- and leisure time related-physical activity. Details of psychosocial factors and depression were inquired using the INTERHEART study questionnaire [27]. The equipments for measurements of height, weight, waist and hip size and blood pressure (BP) were similar at all the centres for ensuring uniformity. Physical examination emphasized measurement of height using stadiometer, weight using calibrated spring weighing machines, waist and hip were measured using spring tapes and sitting BP measured after at least 5 minute rest using Omron SDX (Omron Inc, IL, USA) instruments. Three readings were obtained and averaged for data analyses. Fasting blood sample was obtained from all individuals after 8–10 hours fasting. Samples were collected at community centres by technicians from M/s Thyrocare Technologies Ltd. Laboratory (an accredited national laboratory, www.thyrocare.com). Blood glucose was measured at the local biochemistry facility of these laboratories and serum transported in dry-ice to the national referral laboratory at Mumbai where a uniform protocol was used for measurements. Cholesterol, high density lipoprotein (HDL) cholesterol and triglyceride levels were measured using enzyme-based assays with internal and external quality control (www.thyrocare.com). Values of low density lipoprotein (LDL) cholesterol were calculated as in previous studies [28].

### Diagnostic Criteria

Socioeconomic status was categorized according to education, occupation and self-categorized socioeconomic scale. Educational status was categorized according to the number of years of formal education into three groups: less than 10 years (illiterates and less than secondary), 10–15 years (secondary to graduate) and more than 15 years (postgraduate) as in a previous study [16]. Occupational classes were grouped according to British Social Register and categorized into three groups high (classes 1–2), medium (classes 3 non-manual, 3 manual) and low (classes 4–5) [9]. In case of unemployed housewives, the occupational class of husband was used. Socioeconomic status was identified into 10 groups depending on perceived status and was grouped into high (1–3), medium (4–6) and low (7–10) similar to previous studies [27]. Such classification has been validated in previous epidemiological studies in India and correlate well with income, asset ownership and housing [29]. Smokers included subjects who smoked cigarettes, *bidis*, or other smoked forms of tobacco daily, past smokers were subjects who had smoked for at least 1 year and had stopped more than a year ago. Users of other forms of tobacco (mainly chewed tobacco) were classified as non-smoked tobacco use. The diagnostic criteria for tobacco use as well as other coronary risk factors have been advised by the World Health Organization (WHO) [20]. Subjects consuming more than 20 g visible fat daily were categorized as high fat intake and those consuming <2 servings of fruits or vegetables daily as low intake [30]. Those with no regular work-related or leisure-time physical activity were classified as having physical inactivity, as in earlier studies [21]. Depression was diagnosed using the 3-question matrix according to INTERHEART study [27]. Stress was inquired as work-related, family-related or financial-related stress in last 12 months and presence of any one, two or three variables was classified as mild, moderate and severe stress respectively [27]. Overweight or obesity was defined as body mass index (BMI) ≥25 kg/m^2^ [31].Truncal obesity was diagnosed when waist:hip ratio was >0.9 in men and >0.8 in women or waist circumference was >90 cm in men and >80 cm in women according to the internationally harmonized definition of metabolic syndrome in South Asians [31], [32]. Hypertension was diagnosed when systolic BP was ≥140 mm Hg and/or diastolic BP≥90 mm Hg or a person was a known hypertensive. Dyslipidemia was defined by the presence of high total cholesterol (≥200 mg/dl), high LDL cholesterol (≥130 mg/dl), low HDL cholesterol (<40 mg/dl in men and <50 mg/dl in women) or high triglycerides (≥150 mg/dl) according to National Cholesterol Education Program Adult Treatment Panel-3 (NCEP-ATP-3) guidelines [33]. Diabetes was diagnosed on the basis of either history of known diabetes or fasting glucose ≥126 mg/dl. The diagnosis of the metabolic syndrome was based on the harmonized definition with South Asian criteria for waist size [32]. Accordingly, it was diagnosed when any three of the following five criteria- waist size >90 cm men, >80 cm women; BP systolic ≥130 and/or diastolic ≥85 mm Hg; fasting triglycerides ≥150 mg/dl; HDL cholesterol <40 mg/dl men, <50 mg/dl women; and fasting blood glucose >100 mg/dl or known diabetes- were present.

### Statistical Analyses

All the paper case-report form data were entered into a SPSS database (Version 13.0, SPSS Inc, Chicago, USA). Data for men and women have been analyzed separately. Numerical variables are reported as mean ± 1 SD and categorical variables as percent. Correlation of various socioeconomic parameters (educational level, occupational class and perceived socioeconomic status) was performed using non-parametric Kendall’s test-B and ? calculated. Age- and sex-adjustment was performed for categorical variables using the direct method with 2001 Indian population as standard. Intergroup comparisons were performed using *X* ^2^ test for categorical variables. Multiple group comparisons were also performed by *X* ^2^ for categorical variables. To determine significance of differences in risk factors in various socioeconomic classes, the prevalence of risk factors in the highest educational status, occupational class or socioeconomic group was compared with medium and low groups after age- and sex-adjustment. Odds ratio (OR) and 95% confidence intervals (CI) were calculated using multivariate logistic regression. P value of <0.05 was considered significant.

## Results

The study was performed at 11 cities in India. These cities are located in northern (Jammu, Chandigarh, Bikaner), western (Ahmadabad, Jaipur), southern (Madurai, Belgaum, Nagpur) and eastern (Lucknow, Patna, Dibrugarh) regions. 6198 adult subjects (men 3426, women 2772) of the targeted 9,900 subjects were evaluated (response 62%). Recruitment at individual sites was Ahmadabad 490, Bikaner 499, Belgaum 50, Chandigarh 502, Dibrugarh 500, Jaipur 1324, Jammu 320, Lucknow 835, Madurai 923, Nagpur 264 and Patna 491. More than 90% of all variables were available for analyses. Social and demographic characteristics in men and women are shown in Table 1. Men were slightly older than women and there was no significant difference across various age-groups. Low educational status (illiteracy and <10 years of formal education) was more among women (47.6%) as compared to men (22.3%) and majority of subjects belonged to middle socioeconomic status. More than half of all men and women lived in joint families and 85.6% were married. 17% subjects had migrated from rural-to-urban locations.

**Table 1:** Social and demographic variables of study subjects.

Variables	Men N = 3426	Women N = 2772	P value (X^2^ test)
Age groups			
<30	254 (7.4)	205 (7.4)	1.00
30–39	573 (16.7)	568 (20.5)	0.489
40–49	915 (26.7)	786 (28.4)	0.776
50–59	879 (25.7)	638 (23.0)	0.627
60–69	539 (15.7)	447 (16.1)	1.00
70+	266 (7.8)	128 (4.6)	0.349
Age Mean ± 1 SD (yr)	49.2±13.5	47.3±12.8	<0.001
Educational status			
0–10 years	700 (22.3)	1160(47.6)	0.002
11–15 years	1463(46.7)	881(36.2)	0.132
>15 years	970(31.0)	396(16.2)	0.0137
Occupational class			
Professional	799(24.1)	425(16.0)	0.152
Business	1117(33.6)	676(25.4)	0.203
Manual skilled	849(25.5)	553(20.8)	0.430
Non-manual skilled	136(4.1)	138(5.2)	0.711
Manual labor	83(2.5)	142(5.3)	0.306
Unemployed	338(10.2)	724(27.2)	0.002
Socioeconomic status			
1–3 (high)	182(6.5)	129(5.8)	0.836
4–6 (middle)	1944(69.3)	1678(74.8)	0.386
>6 (low middle and low)	679(24.2)	435(19.4)	0.411
Rural-urban migrants	632(19.0)	421(15.9)	<0.001
Family type			
Nuclear	1156(34.8)	1061(39.1)	0.528
Extended	459(13.8)	321(11.8)	0.672
Joint	1691(50.9)	1298(47.9)	0.671
Others	17(0.5)	31(1.1)	0.633
Marital status			
Married	2973(88.7)	2333(86.7)	1.0
Unmarried	241(7.2)	119(4.4)	0.396
Others	136(4.0)	238(8.8)	0.165

Age-adjusted prevalence of risk factors is shown in Table 2. In men and women, respectively, prevalence of smoking was in 12.0% and 0.5%, other tobacco use in 12.7% and 6.3%, ex-smoking in 5.1% and 0.9%, either smoking or tobacco use in 25.8% and 7.3%, high fat intake ≥20 g/day in 51.2% and 48.2%, low fruit/vegetables intake <2 servings/day in 25.3% and 28.9%, <moderate grade physical activity in 38.8% and 46.1%, depression in 20.0% and 20.9%, moderate stress in 24.1% and 31.5% and severe stress in 14.5% and 13.9%. In men and women, respectively, prevalence of overweight or obesity with BMI ≥25 kg/m^2^ was in 41.1% and 45.2%, obesity BMI ≥30 kg/m^2^ in 8.3% and 15.8%, high waist circumference in 35.7% and 57.5%, high waist-hip ratio in 69.0% and 83.8%, hypertension in 32.5% and 30.4%, high total cholesterol in 24.8% and 25.3%, low HDL cholesterol in 34.1% and 35.1%, high triglycerides in 41.2% and 31.5%, diabetes in 16.7% and 14.4%, and metabolic syndrome in 32.2% and 40.4% subjects respectively. Known cardiovascular disease was in 2.8% men and 2.2% women (Table 2).

**Table 2:** Age-adjusted prevalence of lifestyle and cardiometabolic risk factors in the study cohort.

Variables	Total	Men	Women
	N = 6198	N = 3426	N = 2772
Smoking			
Current smokers	426(6.9)	411(12.0)	15(0.5)
Ex smokers	200(3.2)	174(5.1)	26(0.9)
Other tobacco use	610(9.8)	435(12.7)	175(6.3)
Ever smoking and/or tobacco	1086(17.5)	884(25.8)	202(7.3)
Alcohol consumption			
<7 drinks/week	471(7.6)	429(12.5)	42(1.5)
> = 7 drinks/week	22 (0.3)	22(0.6)	–
Visible fat intake			
<20 g/day	1768(28.5)	903(26.3)	865(31.2)
20–40 g/day	2218(35.8)	1255(36.6)	963(34.7)
>40 g/day	874(14.1)	500(14.6)	374(13.5)
Fruits and vegetables intake≥2 servings/day<2 servings/day	3637(58.7)1670(26.9)	2069(60.3)868(25.3)	1568(56.5)802(28.9)
Moderate physical activity (work or leisure)			
Mild	3077(49.6)	1773(51.7)	1304(47.0)
Moderate	1211(19.5)	66819.5)	543(15.8)
Vigorous	115(1.8)	93(2.7)	22(0.8)
Stress (home, work or financial)			
Mild (any one)	1662(26.8)	1003(29.3)	659(23.8)
Moderate (any two)	1703(27.5)	828(24.1)	875(31.5)
Severe (all three)	883(14.2)	497(14.5)	386(13.9)
Depression	1268(20.4)	687(20.0)	581(20.9)
Obesity			
≥25 kg/m^2^	2660(42.9)	1408(41.1)	1252(45.2)
≥30 kg/m^2^	721(11.6)	283(8.3)	438(15.8)
Truncal obesity			
Waist >90/>80 cm, men/women	2819(45.5)	1224(35.7)	1595(57.5)
WHR >0.9/>0.8, men/women	4690(75.7)	2366(69.0)	2324(83.8)
Hypertension	1957(31.6)	1114(32.5)	843(30.4)
High cholesterol total (≥200 mg/dl)	1551(25.0)	849(24.8)	702(25.3)
High triglycerides (≥150 mg/dl)	2286(36.9)	1411(41.2)	875(31.5)
Low HDL cholesterol (<40 men/<50 women mg/dl)	2141(34.5)	1168(34.1)	973(35.1)
Diabetes (known or fasting glucose ≥126 mg/dl)	973(15.7)	573(16.7)	400(14.7)
Metabolic syndrome	2212(35.7)	1103(32.2)	1109(40.4)
Known cardiovascular disease (CHD)	158(2.5)	96(2.8)	62(2.2)

Numbers in parentheses are percent. BMI body mass index; WHR waist hip ratio; LDL low density lipoprotein; HDL high density lipoprotein; CHD coronary heart disease.

Prevalence of various cardiovascular risk factors in low, middle and high educational, occupational and socioeconomic groups is shown in Table 3. Smoking/tobacco use, smoking as well as non-smoked tobacco use, and any psychosocial stress was more among the low educational, occupational and socioeconomic status subjects while high fat intake more in high educational, occupational and socioeconomic groups. Overweight and obesity was more in high educational, occupational and socioeconomic status subjects, central obesity defined by high waist size was similar while according to high waist-hip ratio was more in the low educational, occupational and socioeconomic subjects. Prevalence of hypertension, hypercholesterolemia, diabetes and metabolic syndrome was similar across the groups while low HDL cholesterol and high triglycerides more in low educational and socioeconomic groups (*X* ^2^ test, p<0.05).

**Table 3:** Age- and sex-adjusted prevalence of risk factors among low, middle and high socioeconomic status groups, occupational classes and educational groups (numbers, %).

Variables	Educational status			Occupational class			Socioeconomic status		
	Low(n = 1248)	Middle(n = 2956)	High(n = 1366)	Low(n = 1287)	Middle(n = 1677)	High(n = 3018)	Low(n = 374)	Middle(n = 3622)	High(n = 1114)
**Lifestyle**									
Smoking/tobacco use	303(24.3)	425(14.4)	260(19.0)	208(16.1)	341(20.3)	504(16.7)	66(17.6)	710(19.6)	173(15.5)
Smoking	77(6.1)	266(9.0)	204(14.9)	66(5.1)	172(10.2)	377(12.5)	36(10.4)	375(10.3)	126(11.3)
Non-smoked tobacco	248(19.9)	235(7.9)	100(7.3)	154(11.9)	218(13.0)	227(7.5)	42(12.1)	445(12.3)	74(6.6)
Alcohol abuse	120(9.6)	305(10.3)	158(11.5)	66(5.1)	224(13.3)	327(10.8)	39(11.2)	421(11.6)	127(11.4)
High dietary fat	577(46.2)	1472(49.8)	819(60.0)	523(40.6)	834(49.7)	1680(55.7)	145(41.8)	1923(53.1)	681(61.6)
Low dietary fruit/vegetable	299(23.9)	868(29.3)	385(28.9)	346(26.9)	450(26.8)	797(26.4)	166(47.8)	893(24.7)	243(21.8)
Low physical activity	464(37.2)	1347(45.5)	485(35.5)	610(47.4)	732(43.7)	1181(39.1)	147(42.3)	1453(40.1)	411(36.9)
Stressful event (any event)	399(32.0)	795(27.0)	278(20.3)	339(26.3)	563(33.6)	716(23.7)	112(29.9)	1085(29.9)	267(23.9)
Depression	226(18.1)	661(22.3)	242(17.7)	217(16.8)	382(22.8)	642(21.3)	72(19.2)	826(22.8)	208(18.7)
**Cardiometabolic**									
BMI≥25kg/m^2^	487(39.0)	1258(42.5)	632(46.2)	457(35.5)	639(38.1)	1473(48.8)	124(35.7)	1510(41.7)	556(49.9)
BMI≥30 kg/m^2^	100(8.0)	339(11.4)	152(11.1)	146(11.3)	145(8.6)	388(12.8)	32(9.2)	408(11.3)	129(11.6)
Waist size >90 men/>80 cm women	500(40.6)	1391(47.0)	689(50.4)	578(44.9)	633(37.7)	1565(50.0)	182(52.4)	1633(45.1)	557(50.0)
Waist-hip ratio >0.9 men/>0.8 women	1029(82.4)	2331(78.8)	899(65.8)	1006(78.1)	1337(79.8)	2224(73.7)	264(76.0)	2856(78.8)	809(72.6)
Hypertension	397(31.8)	874(29.5)	466(34.1)	392(30.4)	487(29.0)	1009(33.4)	94(27.1)	1135(31.3)	370(33.2)
Cholesterol total ≥200 mg/dl	300(24.0)	708(23.9)	373(27.3)	324(25.2)	390(23.2)	790(26.2)	86(24.8)	907(25.0)	262(23.5)
HDL cholesterol <40 men/<50 women (mg/dl)	472(37.8)	973(32.9)	393(28.8)	458(35.6)	620(37.0)	977(32.4)	134(38.6)	1260(34.8)	371(33.3)
Triglycerides ≥150 mg/dl	460(36.8)	1060(35.8)	494(36.1)	427(33.2)	689(41.1)	1063(35.2)	113(32.5)	1386(38.2)	378(33.9)
Diabetes	181(14.5)	452(15.3)	195(14.3)	194(15.1)	260(15.5)	486(16.1)	67(19.3)	549(15.2)	158(14.2)
Metabolic syndrome	441(35.3)	1012(34.2)	473(34.6)	442(34.3)	577(34.4)	1088(36.0)	127(36.5)	1256(34.6)	389(34.9)

BMI body mass index; HDL high density lipoprotein.

**Figure 1:**
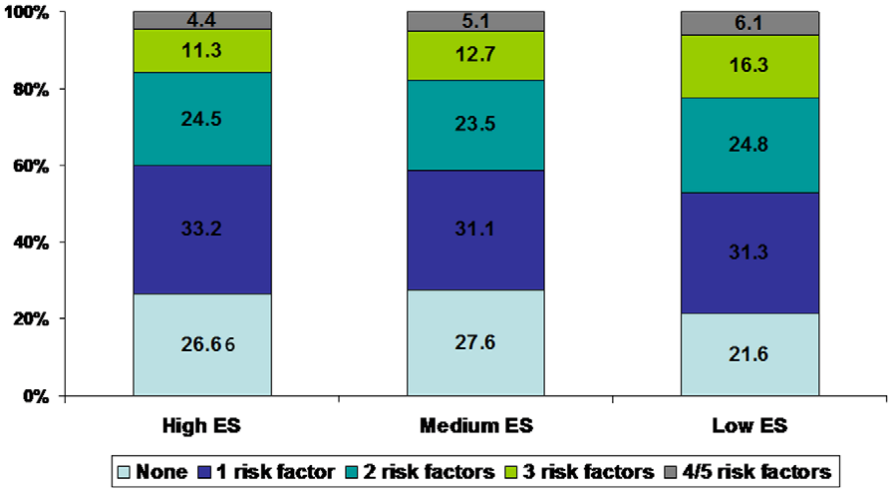
Prevalence of risk factors (smoking/tobacco use, hypertension, hypercholesterolemia, diabetes, and metabolic syndrome) in different educational status (ES) subjects. Prevalence of ≤1 risk factor is significantly greater in high educational status group while prevalence of ≥3 risk factors more in low educational status group (p<0.01).

Prevalence of combination of one or more risk factors (smoking/tobacco use, hypertension, hypercholesterolemia, diabetes and metabolic syndrome) in different educational status groups is shown in Figure 1. Almost a quarter of subjects had no risk factors, least in the low education group (18.6%) as compared to medium (24.4%) and high (23.2%) (p<0.01). Presence of three or more risk factors was significantly greater in low educational status subjects (28.0%) as compared to medium (23.9%, p = 0.004) and high educational status (22.1%) (p<0.001).

**Figure 2:**
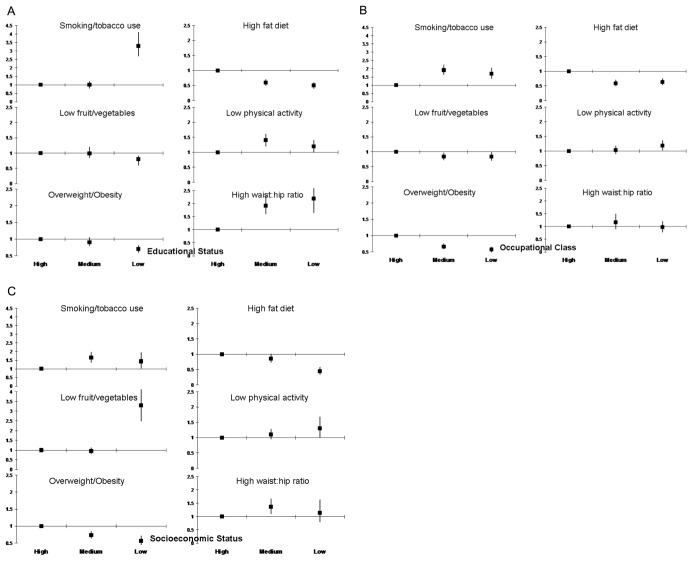
Age- and sex-adjusted odds ratios for prevalence of lifestyle risk factors in high, middle and low educational, occupational and socioeconomic groups. There is significantly greater prevalence of smoking/tobacco use, low physical activity, low fruit and vegetable intake and high waist-hip ratio among the medium and lower status groups as compared to high (p<0.05). Prevalence of high fat dietary intake and overweight/obesity is greater among the higher status group.

**Figure 3:**
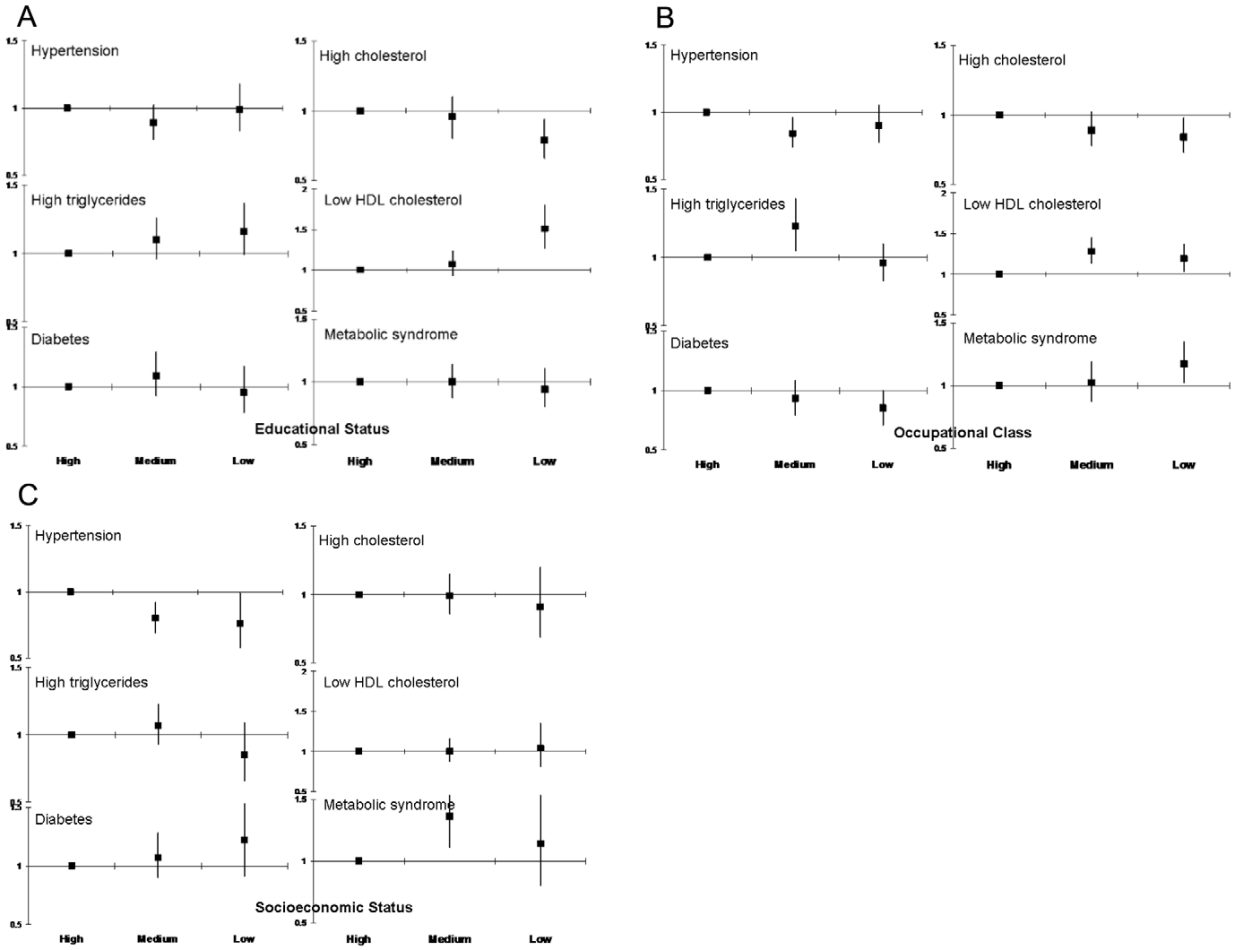
Age- and sex-adjusted odds ratios for prevalence of cardiometabolic risk factors in high, middle and low educational, occupational and socioeconomic groups. There is significantly greater prevalence of hypertriglyceridemia and low HDL cholesterol among the low educational (top panel), occupational (middle panel) and socioeconomic status (lower panel) subjects while metabolic syndrome is more in the low occupational classes (p<0.05). Prevalence of hypercholesterolemia is more in higher groups while that of other risk factors similar.

There was significant correlation of socioeconomic status, occupational class and educational level (Kendall’s T-B, p<0.001). Educational status correlated more strongly with occupation and socioeconomic status as compared to the others in both men and women (Table 4). As compared to high educational status subjects, in low educational status group there was greater prevalence of smoking and/or tobacco use (OR 3.27, 95% CI 2.66–4.01), non-smoked tobacco use (5.53, 4.31–7.09), low physical activity (1.15, 0.97–1.37), high waist:hip ratio (2.18, 1.65–2.71), low HDL cholesterol (1.51, 1.27–1.80) and hypertriglyceridemia (1.16, 0.99–1.37) (Figures 2a and 3a). Prevalence of high fat diet (0.47, 0.38–0.57), overweight/obesity (0.68, 0.58–0.80) and hypercholesterolemia (0.79, 0.66–0.94) was lower in low educational status subjects. In middle educational status subjects there was greater prevalence of non-smoked tobacco use (1.25, 0.99–1.57), physical inactivity (1.37, 1.20–1.58), high waist:hip ratio (1.91, 1.60–2.28) and high triglycerides (1.10, 0.96–1.26) compared to high educational status subjects. Lifestyle and cardiometabolic risk factors in middle and low occupational (Figures 2b and 3b) and socioeconomic classes (Figures 2c and 3c) as compared to the highest were also determined. Associations of abnormal lifestyles and cardiometabolic risk factors were similar to educational status although the magnitude was lower. Smoking/tobacco use, physical inactivity and high-waist hip ratio were more in lower occupational classes and socioeconomic groups (Figure 2b and 2c). Prevalence of low HDL cholesterol and high triglycerides was greater in low occupational and socioeconomic groups while metabolic syndrome was greater in low occupational group (Figure 3b and 3c). The odds for association of educational status with hypertension, diabetes, hypercholesterolemia and metabolic syndrome attenuated after adjustment for lifestyle variables- smoking/tobacco use, dietary fats and fruits/vegetables intake, physical activity and psychosocial stress.

**Table 4:** Age-adjusted correlation of various indices of socioeconomic status (Kendall’s tau-B).

	Education		Occupation		Socioeconomic status	
	Men	Women	Men	Women	Men	Women
Education	–	–	0.35±0.01***	0.31±0.02***	0.15±0.02***	0.15±0.02***
Occupational class	0.35±0.01***	0.31±0.02***	–	–	0.15±0.02***	0.14±0.01***
Socioeconomic status	0.15±0.02***	0.15±0.02***	0.15±0.02***	0.14±0.01***	–	–

*** p<0.001.

## Discussion

This study shows high prevalence of cardiometabolic risk factors in the Asian Indian urban population. Low educational, occupational and socioeconomic status are important markers of risk. Low educational status subjects have greater prevalence of central obesity (high waist-hip ratio), low HDL cholesterol, hypertriglyceridemia, smoking and tobacco use and low physical activity. There is greater clustering of three or more risk factors in low educational status men and women.

Multiple parameters could be used to assess socioeconomic status [34], [35]. These include macrolevel social determinants of health (social organization, the economy, work environment, transport, national and regional human or social development indices, etc.), [33] individual level factors (poverty, stress, life-course social gradient, psychosocial work environment, social support and social cohesion, food and nutrition, social exclusion, social patterning of individual health behaviors, smoking, etc.) [34] and transgenerational, in-utero and early life events, [36] besides the ones used in the present study- education, occupation and socioeconomic scale. Education is a summary measure of early life experiences and higher parental education promotes maternal dietary adequacy and child literacy, leads to better and stable occupation, less chances of unstable migration, better incomes and also provides coping abilities [34]. Educational status has been used the most in cardiovascular epidemiological studies as marker of socioeconomic status as it is stable after early childhood and least influenced by social changes or illness in adulthood [34]. We have previously reported that in India educational level correlates significantly with occupation, housing, neighborhood measures and social status [14]. Similar associations have been reported in other studies from India and elsewhere [37,38[. In the present study also there is significant correlation of educational status with occupation as well as socioeconomic scale (Table 4). We, therefore, used this measure in the present study although correlation of lifestyle and cardiometabolic risk factors with occupational classes and socioeconomic status groups show associations similar to that with educational status.

This study is the first from India that shows greater prevalence of multiple cardiometabolic risk factors such as high waist-hip ratio, low HDL cholesterol and hypertriglyceridemia in low educational status subjects and metabolic syndrome in low occupational class subjects. The risk factors are attenuated after multivariate adjustments for lifestyle factors. This shows that in the lower educational and socioeconomic status subjects unhealthy lifestyles are the major determinants of increased cardiovascular risk. Previous epidemiological studies from India in the middle of twentieth century reported lower cardiovascular risk factors among the poor as compared to high socioeconomic status individuals [11]. Although these studies reported greater prevalence of smoking and tobacco use among low socioeconomic groups, the prevalence of cardiometabolic risk factors was more among the high socioeconomic groups [12], [13]. No studies were available that evaluated cardiovascular mortality among different socioeconomic groups. Studies at turn of the twentieth century reported greater prevalence of smoking, hypertension, and in some studies, hypercholesterolemia among the illiterate and subjects with low socioeconomic (educational) status [14]–[18]. Among apparently homogenous Indian industrial subjects, cardiovascular risk factors such as smoking was more in low educational status subjects while obesity and metabolic risk factors greater in better educated classes [15]. The present study shows that not only smoking and tobacco use, but also multiple cardiometabolic risk factors, are greater among the less educated urban subjects. The odds of prevalence of these risk factors vary from 1.2 to 1.5, which implies 20–50% greater prevalence among the less literate. These differences are similar to studies in high and middle income countries [34], [38].

Multiple factors are associated with greater prevalence of cardiovascular risks among the low socioeconomic status subjects. These include social disorganization, adverse early life events, poor life course social gradient, unemployment, stressful environment at work, poor public transport, low social support and cohesion, unhealthy dietary habits, poverty, social exclusion and health behaviors other than smoking [34], [35], [38], [39]. In India, there could be culturally specific issues such as lack of discourse on health-related issues in children, adolescents and women due to patriarchal dominated social systems, communication and language barriers and poor health literacy [1], [2], [40]. We have not studied many of these factors and cannot comment on their importance. The present study does not show a significantly greater prevalence of depression among the lower socioeconomic status subjects although psychosocial stress is greater in low educational status individuals. Attenuation of odds ratios after adjustment of various lifestyle variables including psychosocial stress is indicative of their importance in explaining educational and socioeconomic differences. Sociologists have suggested that inverse association of cardiovascular risk is not necessarily unidirectional, since health can function as a selective mechanism in relation to socioeconomic status [41], [42]. The Whitehall-II study evaluated causation of inverse association of cardiovascular risk factors with social status. 8312 men and women were followed up for 10 years between 1991 and 2004 for identification of associations of health-related selections vs. social causation in causing cardiovascular risk [43]. It was concluded that social causation contributes to socioeconomic differences in cardiometabolic health in midlife while health-related selection operates at younger ages. Findings of greater prevalence of cardiometabolic risk factors in low educational status subjects in the present study shows that social causation hypothesis is important in causing greater risks. Prospective studies in India are needed to confirm these findings.

There are a number of limitations of this study. Major ones include the sample size and external validity. The sample size may be considered insufficient for a country of size of India, moreover, rural areas have not been studied. However, this is the only study from India that has evaluated more than ten states at different stages of human and economic development [19]. This is also the only study that used a common protocol, case-report forms, measurement techniques and biochemical estimations at a single national laboratory. Moreover, the study was designed to study the urban areas which are currently the hot-spots of cardiovascular epidemic in India [5]. The sample size is considered adequate by World Health Organization to identify risk factors [20]. Secondly, low response rate in the study (62%) is also a concern and it is possible that those excluded were either more or less healthy as compared to the study subjects as we do not have basic demographic data of the non-responders. This is a major study limitation. On the other hand, these response rates are similar to other population based studies in India [5], [19]. Some studies from Western Europe and America have reported response rates as low as 35–40% [44]. There is no scientifically proven minimally acceptable response rate and it has been opined that a response rate of 60% be considered as threshold of acceptability and has face validity as a measure of survey quality [45]. Empirical assessments over the past decade have concluded that the response rate of a survey may not be as strongly associated with the quality of representativeness of the survey as is generally believed [46]. Thirdly, we have not analyzed the “causes of the causes” or the societal factors. Psychosocial factors such as depression and perceived stress could be important mediators of increased cardiovascular risk in low socioeconomic status subjects [47]. Our study shows that psychosocial stress in the last 12 months is greater in low educational and socioeconomic status subjects. We have not analyzed this further as the numbers of questions were limited. It is likely that larger versions of depression and stress questionnaires would provide additional information. Other limitations include potential problems in recruitment discussed above and missing data for some of the variables although for important variables more than 90% data were available. To overcome this we have age-adjusted the data. Subjects with diagnosed cardiovascular disease are very low in the cohort (2.5%, Table 2) and would not influence the results. On the other hand, as noted above, this is the first Indian study with nationwide representation, there are significant numbers of illiterate and low educational status subjects similar to Indian population [25], we have studied multiple parameters to identify socioeconomic status, and we have evaluated multiple cardiovascular risk factors and their lifestyle determinants.

Rapidly expanding Indian economy has led to large inequities in incomes, education and socioeconomic status [1]. Such inequalities lead to poor health status [48]. Whole populations are pushed into poverty by ill health, especially in low-income countries [49]. In USA and Western European countries cardiovascular mortality and cardiovascular risk factors have declined significantly in high socioeconomic and educational groups while there is no change, even an increase, in the lower socioeconomic status subjects [50], [51]. Whether similar trends emerge in India and other low income countries needs to be evaluated in prospective studies.

In conclusion, this study shows that in Asian Indian urban subjects socioeconomic factors, especially educational status, are important markers of cardiovascular risk. Low educational status is associated not only with greater smoking and tobacco use and lower intake of fruits and vegetables, but, also with lower physical activity and greater prevalence of central obesity (high waist-hip ratio), low HDL cholesterol and hypertriglyceridemia. All these are important markers of cardiovascular risk. An important finding of the present study is attenuation of multiple cardiovascular risk factors after adjustment for unhealthy lifestyles. This shows that promotion of physical activity, more fruits and vegetable consumption, restriction of dietary fats, smoking and tobacco cessation, and stress management are the ways ahead for prevention of cardiovascular diseases among all socioeconomic groups in India.
